# IMU Sensor-Based Hand Gesture Recognition for Human-Machine Interfaces

**DOI:** 10.3390/s19183827

**Published:** 2019-09-04

**Authors:** Minwoo Kim, Jaechan Cho, Seongjoo Lee, Yunho Jung

**Affiliations:** 1 School of Electronics and Information Engineering, Korea Aerospace University, Goyang-si 10540, Korea (M.K.) (J.C.); 2 Department of Information and Communication Engineering, Sejong University, Seoul 143-747, Korea

**Keywords:** dynamic time warping (DTW), hand gesture recognition (HGR), inertial measurement unit (IMU), machine learning, real-time learning, restricted coulomb energy (RCE) neural network

## Abstract

We propose an efficient hand gesture recognition (HGR) algorithm, which can cope with time-dependent data from an inertial measurement unit (IMU) sensor and support real-time learning for various human-machine interface (HMI) applications. Although the data extracted from IMU sensors are time-dependent, most existing HGR algorithms do not consider this characteristic, which results in the degradation of recognition performance. Because the dynamic time warping (DTW) technique considers the time-dependent characteristic of IMU sensor data, the recognition performance of DTW-based algorithms is better than that of others. However, the DTW technique requires a very complex learning algorithm, which makes it difficult to support real-time learning. To solve this issue, the proposed HGR algorithm is based on a restricted column energy (RCE) neural network, which has a very simple learning scheme in which neurons are activated when necessary. By replacing the metric calculation of the RCE neural network with DTW distance, the proposed algorithm exhibits superior recognition performance for time-dependent sensor data while supporting real-time learning. Our verification results on a field-programmable gate array (FPGA)-based test platform show that the proposed HGR algorithm can achieve a recognition accuracy of 98.6% and supports real-time learning and recognition at an operating frequency of 150 MHz.

## 1. Introduction

A human-machine interface (HMI) presents information to a user regarding the state of a process, accepts commands, and operates associated devices [1]. HMIs designed for human convenience are aimed at allowing users to freely control devices via simple operations without requiring the user’s full attention [2]. Therefore, hand gesture recognition (HGR) is an essential feature of HMIs because it allows users to efficiently control devices with simple hand gestures. HGR gets rid of the limitations of controlling devices, and has been widely used in various fields, such as switching TV channels by drawing numbers in a smart home or turning on the air conditioner with a simple gesture while driving.

HGR can be categorized into vision-based gesture recognition (VGR) and sensor-based gesture recognition (SGR) [3]. VGR is a method of recognizing gestures using camera images, and various technologies have been proposed [4,5,6]. However, VGR accuracy degrades in light-sensitive application scenarios because camera images are affected by lighting conditions [7]. On the other hand, SGR methods have relatively few limitations because they use various sensors that are not affected by lighting conditions such as inertial measurement unit (IMU) sensors, electromyography (EMG) sensors, brain wave sensors, electrocardiograph sensors, and radar sensors [8,9]. Among them, IMU sensors, which are generally compact, are widely used for SGR owing to their low cost and low power characteristics [10]. In addition, because IMU sensors can be directly attached to the user’s body, they can obtain relatively accurate hand gesture data.

Dynamic time warping (DTW) [11,12,13,14], multilayer perceptrons (MLPs) [7,15] and convolutional neural networks (CNNs) [16,17] are widely used to recognize hand gestures with IMU sensors. DTW-based recognition algorithms output the most similar hand gestures by measuring DTW distance between the input data and the representative data of each hand gesture, called templates. Before calculating the distance, DTW is used to align two datapoints via warping with a nonlinear method to make them match each other. Because of this alignment process, these algorithms can cope with deformations in time-dependent data caused by different speeds [18]. DTW-based recognition algorithms exhibit good performance for HGR. However, recognition performance greatly depends on the quality of the selected templates. Therefore, because these algorithms require finding optimal templates from a prepared dataset, it is hard to perform real-time learning with high accuracy, which is one of the most important features for HGR because users want devices to learn their hand gestures immediately. MLPs calculate the weighted sum of input features and are used in many studies because of their high recognition performance [7,15]. The learning algorithm of MLPs uses the gradient descent method to find the optimal weights that minimize recognition errors. These algorithms have a high computational complexity, which makes it difficult to train them in real time. As MLPs become deeper to achieve higher performance, the number of weights increases exponentially, and their computational complexity becomes much higher. In addition, MLPs are vulnerable to the deformation of the input data. To cope with this problem, researchers have come up with three ideas involving CNNs: local receptive fields, shared weights, and spatial subsampling [19]. In CNNs, raw sensor data are used without extracting any features, which represents an advantage in that no data are lost through feature extraction processing [16]. Some studies have demonstrated superior performance in HGR using CNNs, but it is hard to train such network in real time because their learning algorithm is as computationally complex as those of MLPs.

On the contrary, restricted coulomb energy (RCE) neural networks employ a relatively simple training method [20] and can thus perform real-time learning [21]. An RCE neural network consists of neurons, and each neuron has a center point and a radius, which forms an activation region. The training process involves determining the center point of each neuron and changing the size of its radius. In the recognition process, distance information between the center point of each neuron and the input feature data are used to determine whether each neuron is activated. Then, the label of the activated neuron that best matches the input data becomes the recognition result. However, RCE neural networks do not consider the characteristics of time-dependent data when calculating distance information. Therefore, it is hard for an existing RCE neural network to provide high performance in IMU sensor-based HGR.

In this paper, we address the following question: *how can we make a high-performance HGR algorithm that supports real-time learning?* We propose an efficient HGR algorithm that uses a simple learning algorithm based on an RCE neural network and employ the distance measurement method of DTW. To achieve our goal and deliver high-performance gesture recognition, the distance measurement method used in RCE neural networks is removed and that of DTW is employed. A test platform was constructed to verify the real-time operation of the training and recognition algorithms. This platform consisted of an IMU sensor, an Arduino module for data preprocessing, and a field-programmable gate array (FPGA), on which the proposed HGR algorithm was implemented. Performance evaluation results show that the proposed system can achieve an accuracy of 98.6% with a training time of 0.423 ms and a recognition time of 0.426 ms.

The rest of this paper is organized as follows: Section 2 describes the background of RCE neural networks and DTW. Section 3 presents the proposed HGR algorithm, and Section 4 presents the test platform along with its hardware structure. In Section 5, we present the experimental results of the proposed HGR algorithm. Finally, Section 6 concludes the paper.

## 2. Backgrounds

### 2.1. RCE Neural Network

**Figure 1:**
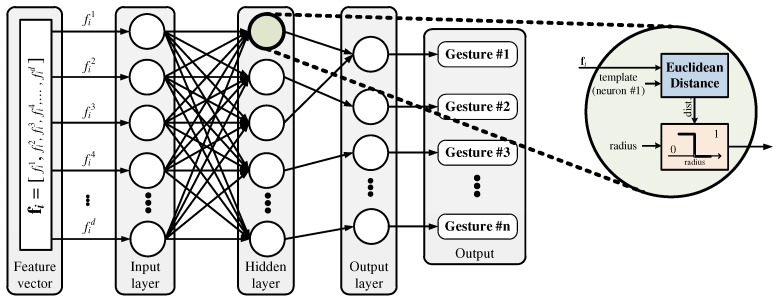
Structure of an RCE neural network.

An RCE neural network consists of three layers: an input layer, a hidden layer, and an output layer, as shown in Figure 1 [22]. The input layer contains feature data and is connected in parallel to each neuron in the hidden layer. Each neuron has a center point and a radius, which forms an activation region similar to the shape of a sphere. In the feature space, the activation region creates a decision boundary to distinguish the data. As the data enter the input layer, each neuron calculates the distance between the input data and its center point. The distance is compared with the radius of each neuron to determine if that neuron is activated, and the results are passed to the output layer. The output layer uses the received information to output the label of the neuron that best matches the input data.

The hidden layer consists of a set of neurons:

(1) $$N=[n_{1},n_{2},\ldots ,n_{j},\ldots ,n_{q}],$$

where *q* represents the total number of neurons generated during the training process. Each neuron can be described by:

(2) $$n_{j}=\left[c_{j},r_{j}\right],$$

(3) $$c_{j}=\left[c_{j}^{1},c_{j}^{2},\ldots ,c_{j}^{d}\right],$$

where j∈{1,2,3,…,q} is the index of each neuron, $c_{j}$ denotes a center point with *d* dimensions, and $r_{j}$ represents the radius of the neuron. If the number of *d*-dimensional feature vectors used in the training process is *m*, the feature vector set can be expressed as follows:

(4) $$F=[f_{1},f_{2},\ldots ,f_{i},\ldots ,f_{m}].$$

The *i*-th feature vector $f_{i}$ is represented as follows:

(5) $$f_{i}=\left[f_{i}^{1},f_{i}^{2},\ldots ,f_{i}^{d}\right].$$

The feature vector is sequentially given as input to each neuron $n_{j}$, and the Euclidean distance $ED(f_{i},c_{j})$ between the feature vector and the neuron center point can be obtained as follows:

(6) $$ED\left(f_{i},c_{j}\right)=\sqrt{{\left(f_{i}^{1}-c_{j}^{1}\right)}^{2}+{\left(f_{i}^{2}-c_{j}^{2}\right)}^{2}+\ldots +{\left(f_{i}^{d}-c_{j}^{d}\right)}^{2}}.$$

Afterwards, the activation of each neuron $n_{j}$ is determined by:

(7) $$ED\left(f_{i},c_{j}\right)\leq r_{j}.$$

If no neurons activated for an input vector $f_{i}$, a new neuron $n_{q+1}$ with a center point $f_{i}$ and an initial radius is generated, and the total number of neurons *q* is increased by one. On the other hand, if there is an activated neuron, there are two possible cases: the label of the activated neuron is the same as the label of the input feature or not. If the two labels are the same, no new neurons are generated. If not, the radius of the activated neuron is reduced to $ED(f_{i},c_{j})$ and a new neuron with a radius of $ED(f_{i},c_{j})$ and a center point equal to the input feature vector $f_{i}$ is created.

### 2.2. DTW

**Figure 2:**
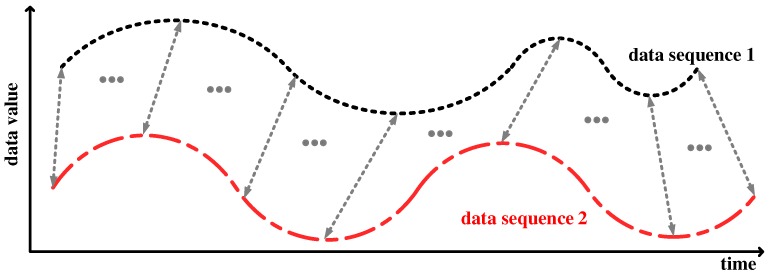
Time alignment of two data sequences; the aligned points are denoted by the arrows.

DTW is a technique for aligning two data sequences by warping them in a nonlinear fashion to match them each other [18], as shown in Figure 2, and then finding distance between the data. This method is widely used in areas that deal with data that changes over time because it results in better performance than employing Euclidean distance on time-dependent data [23].

DTW distance is calculated by finding the optimal alignment between two data sequences, calculating the difference between aligned points, and accumulating the difference values [24]. The problem of finding the optimal alignment can be solved by using an accumulated cost matrix. To obtain this matrix, we need to find the cost matrix in advance. Consider two data sequences:

(8) $$x=\left(x_{1},x_{2},\ldots ,x_{d_{1}}\right),\;y=\left(y_{1},y_{2},\ldots ,y_{d_{2}}\right).$$

**Figure 3:**
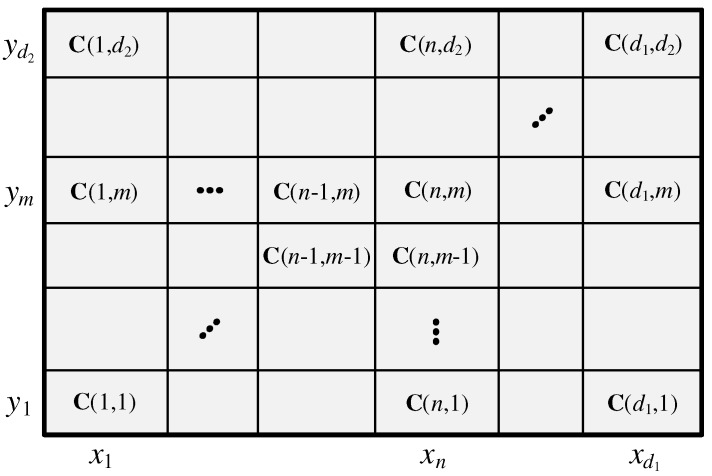
Cost matrix.

The lengths of data sequences x and y are $d_{1}$ and $d_{2}$, respectively. We can find a cost matrix $C\left(n,m\right)\in R^{d_{1}\times d_{2}}$, where $1\leq n\leq d_{1},1\leq m\leq d_{2}$, as shown in Figure 3, by using the following equation:

(9) $$C\left(n,m\right)=|x_{n}-y_{m}|.$$

With this cost matrix, our goal is to find the optimal alignment, which has the minimal overall cost. The optimal alignment is determined by following a small value in the cost matrix. The accumulated cost matrix can be obtained by accumulating the minimum value of the cost matrix under three conditions: the boundary condition, the monotonicity condition, and the step size condition [25]. The optimal path *p* with length *L* can be defined as:

(10) $$p=(p_{1},\ldots ,p_{l},\ldots ,p_{L}),$$

(11) $$p_{l}=\left(n_{l},m_{l}\right)\in C\left(n,m\right)\;\mathrm{for}\;l\in \left[1,L\right].$$

For the optimal path *p*, the following three conditions are defined:

1. Boundary condition
  In the optimal path, the starting point and ending point are defined as:
  (12) $$p_{1}=\left(1,1\right)\;\;\mathrm{and}\;\;p_{L}=\left(d_{1},d_{2}\right).$$
2. Monotonicity condition
  In the optimal path, the index value must be equal to or greater than previous index value:
  (13) $$n_{1}\leq n_{2}\leq \ldots \leq n_{L-1}\leq n_{L}\;\;\mathrm{and}\;\;m_{1}\leq m_{2}\leq \ldots \leq m_{L-1}\leq m_{L}.$$
3. Step size condition
  In the optimal path, the difference between neighboring values has a step size, which can be expressed as the following condition:
  (14) $$p_{l+1}-p_{l}\in \left\{\left(1,0\right),\left(0,1\right),\left(1,1\right)\right\}\;\;\mathrm{for}\;\;l\in \left[1:L-1\right].$$

The formula for the accumulated cost matrix $A\left(n,m\right)\in R^{d_{1}\times d_{2}}$, where $1\leq n\leq d_{1},1\leq m\leq d_{2}$ can be expressed as:

(15) $$A\left(n,m\right)=\left\{\begin{array}{cc} \;C(n,m), & \mathrm{if}\;n=1\;\;\mathrm{and}\;\;m=1,\\ \;A(n-1,m)+C(n,m), & \mathrm{if}\;n>2\;\;\mathrm{and}\;\;m=1,\\ \;A(n,m-1)+C(n,m), & \mathrm{if}\;n=1\;\;\mathrm{and}\;\;m>2,\\ \;\min\{A(n-1,m-1),A(n-1,m),A(n,m-1)\}+C(n,m), & \mathrm{if}\;n>2\;\;\mathrm{and}\;\;m>2. \end{array}\right.$$

**Figure 4:**
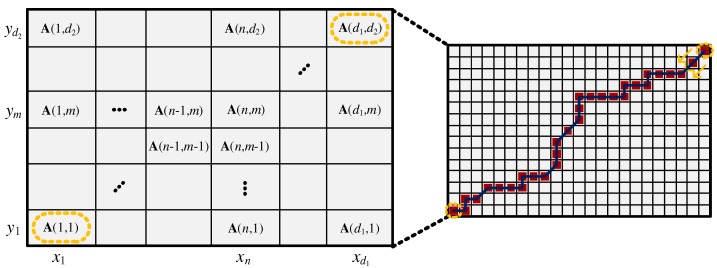
Accumulated cost matrix.

After constructing the accumulated cost matrix, we can find an optimal path by following a small value from $A(d_{1},d_{2})$ to A(1,1), as shown by the dark blue line in Figure 4. The set of index values of the optimal path corresponds to the aligned points shown in Figure 2, and the DTW distance can be expressed as:

(16) $$DTW\left(x,y\right)=A\left(d_{1},d_{2}\right).$$

DTW-based recognition algorithms measure this distance between the input data and the template of each label and output the most similar label.

## 3. Proposed HGR Algorithm

**Figure 5:**
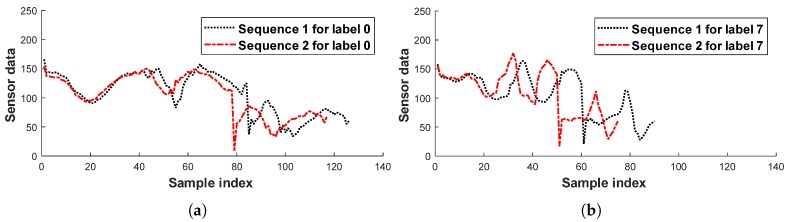
Examples of the data sequences: (**a**) two sequences for label 0; (**b**) two sequences for label 7.

DTW is a nonlinear method for obtaining the distance between two data sequences. The calculation of these distances involves an alignment process that extends or shrinks one data sequence along the time axis to match the other sequence. Thanks to this alignment process, the DTW distance between the two IMU sensor data sequences shown in Figure 5 is more accurate than the Euclidean distance.

**Figure 6:**
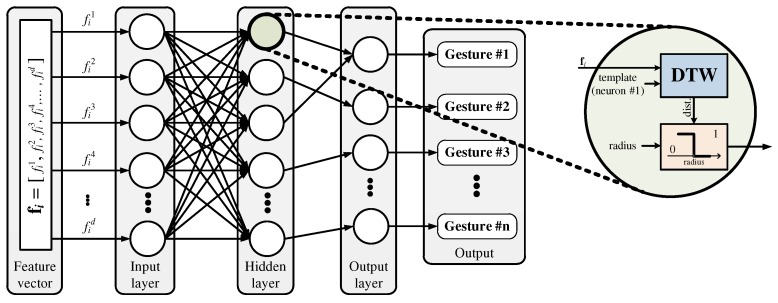
Structure of the proposed HGR algorithm.

This more accurate distance has led to DTW being used in various fields, and, when employed for HGR, it has exhibited superior performance according to many previous studies [11,12,13,14]. However, because DTW-based HGR involves a complex learning algorithm, its applications are limited by the number of recognizable hand gestures. Unlike DTW-based recognition algorithms, RCE neural networks have simple learning algorithms, which allows for real-time training. In other words, they can be used to train the network with the required hand gestures immediately. However, it is hard to use conventional RCE neural networks because they employ the Euclidean distance in their distance measurements during the learning and recognition processes. Therefore, we propose employing DTW in an RCE neural network to create a high-performance HGR algorithm that supports real-time learning. The structure of the proposed HGR algorithm is shown in Figure 6.

Algorithm 1 describes the proposed learning algorithm. When the input feature vector $f_{i}$ is sequentially inputted to each neuron, the distance between the center point of the neuron and the input features is obtained via DTW. Afterwards, whether neuron $n_{j}$ is activated or not is determined by comparing the distance $DTW(f_{i},c_{j})$ with the radius $r_{j}$ of each neuron. The rest of the learning algorithm is the same as that used in existing RCE neural networks. Algorithm 2 describes the recognition process. DTW is applied to obtain the distance between the centers of neurons and the feature data extracted from the hand gesture, and the label of the neuron with the minimum distance becomes the recognition result.

**Algorithm 1** Learning algorithm.

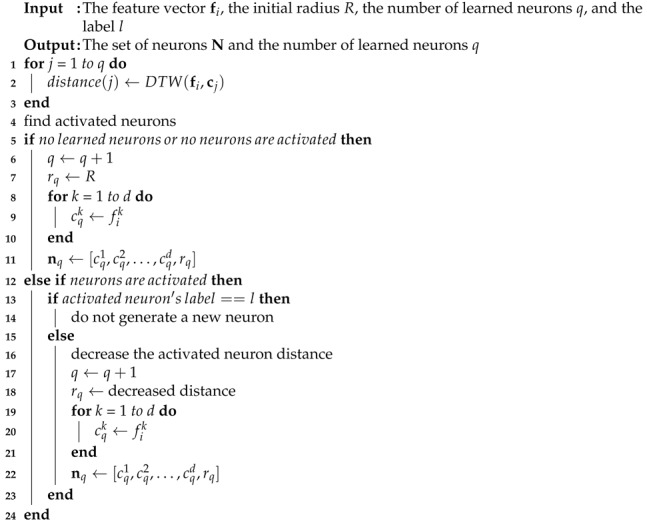



**Algorithm 2** Recognition algorithm.

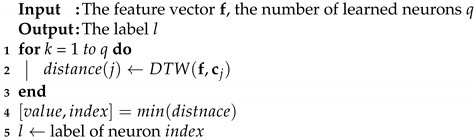



## 4. Test Platform

**Figure 7:**
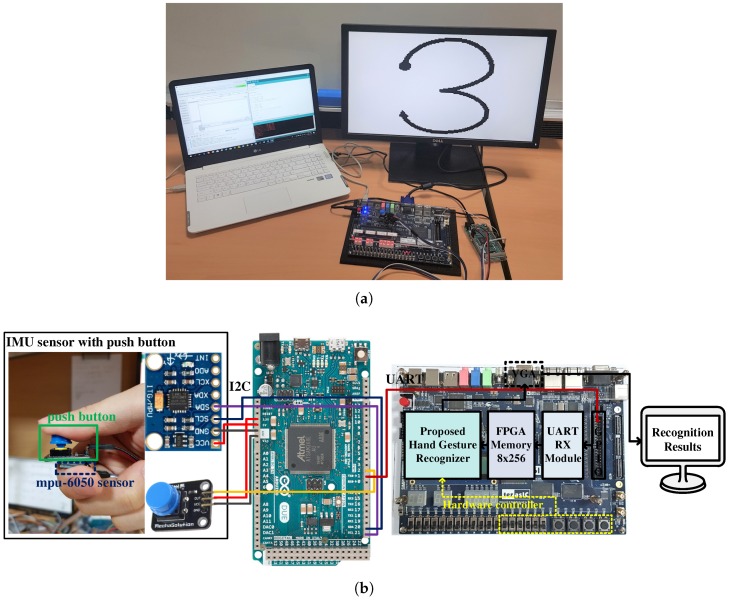
HGR test platform: (**a**) photograph of the test platform; (**b**) configuration of the test platform.

**Figure 8:**
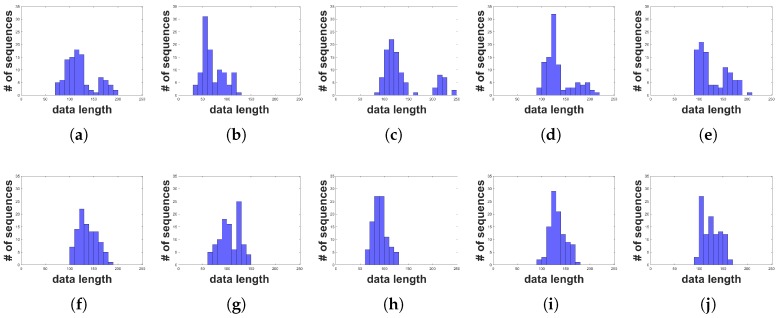
Histogram of the dataset: (**a**) label 0; (**b**) label 1; (**c**) label 2; (**d**) label 3; (**e**) label 4; (**f**) label 5; (**g**) label 6; (**h**) label 7; (**i**) label 8; (**j**) label 9.

We constructed a test platform to evaluate the proposed HGR algorithm, as shown in Figure 7. The platform consisted of an IMU sensor for obtaining hand gesture data, an Arduino module for preprocessing, and an FPGA for the proposed HGR implementation. We used an MPU-6050 [26] as the IMU sensor module, which can measure three-axis acceleration and three-axis gyroscope values. Among these values, we only used three-axis acceleration data for HGR, whereas the gyroscope data were reserved for future use. Hand gesture data from the IMU sensor are transferred to the Arduino through an inter-integrated circuit (I2C) interface by pressing a button on the IMU sensor module. The Arduino module preprocesses the sensor data to make them a fixed length, and a DUE model [27] was chosen considering the memory size and processing speed required for real-time operation. The length of the data depends on the speed and shape of the gesture, and thus data sequences with various lengths need to be processed to make them have a fixed length. We determined that the shortest data length was 33 and the longest one was 243 through experimental tests, as shown in Figure 8. Therefore, we set the fixed data length to 252, which is greater than 243, so that users can freely perform hand movements. Data sequences shorter than 252 are zero padded to avoid affecting the calculation of the distance between data sequences. Preprocessed data are transferred to the FPGA through universal asynchronous receiver/transmitter (UART) communication. The hand gesture recognizer implemented on the FPGA performs the training and recognition functions, and the recognition result is displayed on a monitor.

**Figure 9:**
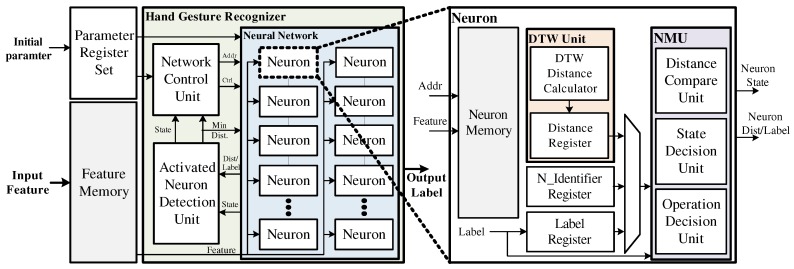
Block diagram of the proposed hand gesture recognizer.

The proposed hand gesture recognizer was designed using Verilog hardware description language (HDL) and implemented in an Intel-Altera Cyclone IV FPGA device to verify that real-time learning and recognition was possible. Figure 9 shows a block diagram of the proposed hand gesture recognizer, which consisted of a neural network, a network control unit (NCU), and an activated neuron detection unit (ANDU). Each neuron consisted of a neuron memory for storing the center point, a DTW unit for calculating the distance, and a neuron management unit (NMU) for managing the state and the operation of the neuron. During the training process, each neuron stores the input features in the memory, and the distance calculation is performed by the DTW unit. The calculated distance and the label information are sent to the ANDU, which finds activated neurons and sends the state of neurons and the minimum DTW distance to the NCU. Using this information, the NCU controls the neural network and modifies the network structure by creating new neurons and modifying the activation regions. During the recognizing process, all the learned neurons compute the DTW distance between the input features and the center point stored in the memory of the learned neurons. Then, the activated neurons transmit their distance to the ANDU, which finds the minimum distance and sends it to the NCU. The NCU then uses the minimum distance to generate a control signal so that the neural network can output the recognition result.

Our FPGA implementation results show that the proposed hand gesture recognizer can be implemented with 30.54 K logic elements and 274.31 Kbits memory as shown in Table 1. In addition, we confirmed that the real-time training and recognition were possible because the proposed hand gesture recognizer required only 0.423 ms for training and 0.426 ms for recognition, at an operating frequency of 150 MHz.

## 5. Experimental Results

**Figure 10:**
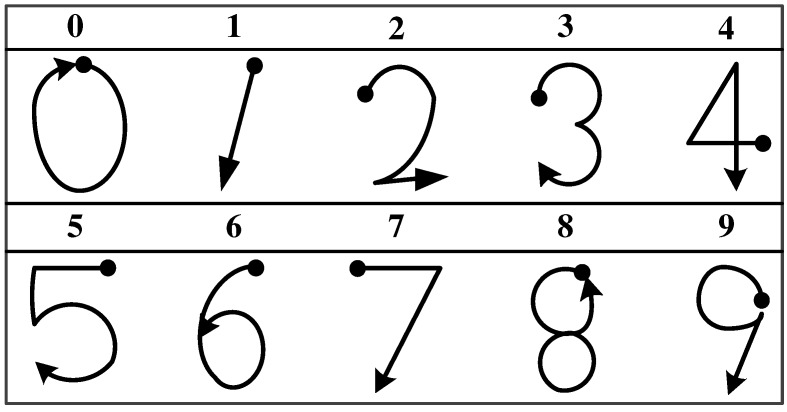
3D number dataset.

We constructed a 3D number dataset similar to the one used in a previous study [11]. Five participants, consisting of four men and one woman, were asked to hold the IMU sensor to write ten digits in the air. The dataset was generated by extracting the accelerometer values at a sampling rate of 20 Hz, and each hand gesture performed from a starting point by following the direction of the arrows shown in Figure 10. Each participant wrote each digit 20 times, and data corresponding to a total of 1000 hand gestures were collected.

Performance evaluation was performed using the collected dataset; accuracy was measured by performing 5-fold cross-validation for each person. Table 2 shows the confusion matrix of the proposed HGR algorithm, and the average recognition accuracy was 98.6%.

Table 3 shows the comparison results in terms of recognition accuracy between existing HGR algorithms and the proposed approach. The RCE neural network in Table 3 performs recognition based on Euclidean distance, and MLP calculates the weighted sum of input features to recognize hand gestures. The DTW-based HGR proposed in [11] recognizes hand gestures based on the DTW distance measurement method as described in Section 2.2. We employed the hand gestures recorded from five participants, and the evaluation results were obtained by 5-fold cross-validation. As shown in this table, the proposed HGR algorithm outperformed the others for all users because it uses the DTW distance measurement method suitable for a time-dependent data sequence such as a hand gesture from an IMU sensor.

## 6. Conclusions

Hand gesture recognition requires the classification of different hand motions depending on the user’s preference or the type of application being considered. However, the application of existing HGR algorithms such as DTW, MLP and CNN is limited because they can only recognize predetermined gestures through preliminary training due to the very complex learning process. In this paper, we proposed an efficient HGR algorithm that can be used in various applications owing to the real-time learning. In order to enable real-time learning with high accuracy, the proposed HGR algorithm uses the learning method of RCE neural networks and distance measurement scheme of DTW. We constructed a test platform with an IMU sensor to verify that real-time learning and recognition was possible using the proposed algorithm. In addition, a 3D number dataset was constructed using the test platform, which could generate three-axis acceleration data samples at 20 Hz. We carried out a performance evaluation using 5-fold cross-validation on the constructed dataset and found that the proposed HGR algorithm could achieve a recognition accuracy of 98.6%, which is 13.2%, 10.6%, and 4% higher than that of RCE neural networks, MLPs, and DTW-based HGR algorithms, respectively. The proposed algorithm was designed and verified in hardware, which could support real-time learning and recognition at an operating frequency of 150 MHz.

With the rapid increase of wearable devices applying HGR technology, the simplified HGR algorithm is required to implement low-cost and low-power very large scale integrated circuits (VLSI). Therefore, our future research is focused on the simplification of the proposed HGR algorithm and its VLSI implementation.

## Figures and Tables

**Table 1:** Implementation results of the proposed hand gesture recognizer.

Block	Neural Network	NCU	ANDU	Total
FPGA Logic Elements (/114,480)	25,765	2540	2970	31,275 (27.32%)
Memory [bists] (/3,981,312)	280,896	0	0	280,896 (7.06%)

**Table 2:** Confusion matrix of the proposed algorithm.

Answer	Prediction									
	0	1	2	3	4	5	6	7	8	9
0	96%	0%	3%	0%	0%	0%	0%	0%	1%	0%
1	0%	100%	0%	0%	0%	0%	0%	1%	0%	0%
2	0%	0%	97%	0%	0%	0%	0%	0%	0%	0%
3	0%	0%	0%	99%	0%	1%	0%	0%	0%	0%
4	0%	0%	0%	0%	100%	0%	0%	0%	0%	0%
5	0%	0%	0%	0%	0%	97%	0%	0%	0%	0%
6	2%	0%	0%	1%	0%	2%	100%	0%	0%	0%
7	2%	0%	0%	0%	0%	0%	0%	99%	0%	0%
8	0%	0%	0%	0%	0%	1%	0%	0%	99%	1%
9	0%	0%	0%	0%	0%	1%	0%	0%	0%	99%
**Total**	100%	100%	100%	100%	100%	100%	100%	100%	100%	100%

**Table 3:** Recognition performance of the proposed algorithm and others.

Algorithm	User1	User2	User3	User4	User5	Average
RCE neural network	82.5%	88.0%	81.0%	92.5%	83.0%	85.4%
MLP	81.5%	91.5%	86.5%	91.0%	89.5%	88.0%
DTW-based HGR	94.6%	94.6%	94.6%	94.6%	94.6%	94.6%
Proposed	99.5%	97.0%	97.5%	99.5%	99.5%	98.6%
